# Intravenous thrombolysis in acute ischemic stroke after recent direct oral anticoagulant intake: toward evidence-informed care

**DOI:** 10.3389/fneur.2026.1904486

**Published:** 2026-08-06

**Authors:** Matija Zupan, Mišo Šabovič, Pawel Kermer, Senta Frol

**Affiliations:** 1 Department of Vascular Neurology, University Medical Centre Ljubljana, Ljubljana, Slovenia; 2 Faculty of Medicine, University of Ljubljana, Ljubljana, Slovenia; 3 Department of Vascular Disorders, University Medical Center Ljubljana, Ljubljana, Slovenia; 4 Department of Neurology, University Medical Center Göttingen, Göttingen, Germany; 5 Department of Neurology, Friesland Kliniken GmbH, Sande, Germany

**Keywords:** acute ischemic stroke, contemporary data, direct oral anticoagulant, idarucizumab, intravenous thrombolysis, Perspective

## Abstract

The use of intravenous thrombolysis (IVT) in acute ischemic stroke (AIS) patients with recent direct oral anticoagulant (DOAC) intake remains one of the most debated issues in contemporary stroke medicine. Current international guidelines generally discourage IVT within 48 h of DOAC ingestion, largely because of concerns regarding symptomatic intracranial hemorrhage (sICH) and the absence of randomized evidence. However, an increasing body of evidence from international registries and multicenter cohorts has not identified a clear increase in sICH among carefully selected patients with recent DOAC exposure who received IVT. Some observational studies have also reported an association with more favorable functional outcomes than those observed in otherwise eligible patients in whom reperfusion therapy was withheld, although these comparisons are susceptible to selection bias and confounding by indication. In this Perspective, we discuss recent advances that have challenged the traditional practice of broadly excluding patients with recent DOAC exposure from IVT. We review observational evidence concerning IVT in selected patients, examine biological hypotheses that could be relevant to the observed clinical findings, and discuss evolving roles of reversal agents and laboratory assessment of anticoagulant activity. We further highlight current implementation barriers and future research priorities, including prospective studies, improved point-of-care anticoagulant testing, and refinement of patient-selection strategies. We argue that contemporary evidence supports reconsideration of rigid time-based exclusion criteria in favor of more individualized, evidence-informed decision-making. Although important uncertainties remain, recent DOAC exposure may not necessarily preclude IVT in appropriately selected patients, particularly when anticoagulant activity can be reliably assessed.

## 1 Introduction

One of the most persistent dilemmas in contemporary stroke care is whether intravenous thrombolysis (IVT) can be safely administered in patients with acute ischemic stroke (AIS) and recent intake of direct oral anticoagulants (DOACs). A recent registry-based analysis did not identify an excess risk of symptomatic intracranial hemorrhage (sICH) among carefully selected patients who received IVT despite recent DOAC exposure and reported an association between IVT and more favorable functional outcomes compared with withholding reperfusion therapy (1). Nevertheless, the observational design leaves these findings susceptible to selection bias, residual confounding, and confounding by indication. Although observational data allow for residual confounding, these findings add to an expanding body of clinical evidence suggesting that recent DOAC use may not invariably preclude IVT in appropriately selected patients.

Current European and American guidelines continue to discourage IVT in patients with recent DOAC ingestion (i.e., within 48 h), due to presumed bleeding risk (2). However, these recommendations are based largely on expert consensus and very low-certainty evidence rather than direct demonstration of harm. The American Heart Association/American Stroke Association permits consideration of IVT when coagulation parameters and renal function are normal (3), whereas the European Stroke Organisation acknowledges significant uncertainty regarding laboratory assessment of DOAC activity and the role of reversal strategies prior to thrombolysis (2). The Australian and New Zealand guidelines recommend idarucizumab administration before IVT in dabigatran-treated patients, although this recommendation is likewise based predominantly on expert opinion rather than randomized evidence (4). For factor Xa inhibitors, guidelines generally advise against routine reversal with andexanet alfa and recommend avoiding IVT within 48 h of ingestion (2).

The discrepancy between increasingly reassuring observational evidence and persistently conservative guideline recommendations raises an important question: Should recent DOAC exposure continue to serve as a broadly applied reason for withholding IVT, or could selected patients be considered for treatment following individualized assessment?

In this Perspective, we examine whether the traditional practice of broadly excluding patients with recent DOAC exposure from IVT remains justified by available evidence. We believe that emerging observational data, coupled with evolving understanding of anticoagulant pharmacology and advances in stroke systems of care, support a transition toward individualized treatment decisions guided by estimated anticoagulant activity rather than fixed time-based exclusion criteria.

## 2 Current advances challenging conventional practice

### 2.1 Accumulating clinical evidence

The management of AIS in patients receiving DOAC therapy may be undergoing a gradual shift. Registry-based and multicenter studies (1, 5, 6) have generally found no signal of increased sICH among selected patients receiving IVT and have reported associations with more favorable functional outcomes compared with withholding treatment. However, these comparisons remain susceptible to selection bias and confounding by indication. In a recently published international SITS-ISTR registry analysis of 258,589 IVT-treated AIS patients, 510 were on dabigatran and 156 received idarucizumab reversal prior to IVT. After propensity-score matching, no statistically significant excess risk was observed among patients receiving dabigatran reversal followed by IVT compared with patients without prior oral anticoagulant use, with broadly comparable rates of any parenchymal hematoma (3% vs. 8.6%), sICH (SITS-MOST: 1% vs. 0.7%), 3-month mortality (25.4% vs. 18.9%), and functional independence (mRS 0–2: 50.9% vs. 52.3%) (7). Moreover, a secondary matched comparison of dabigatran-treated patients receiving IVT with vs. without idarucizumab also found no significant differences in hemorrhage, mortality, or functional outcome.

Similarly, Meinel et al. found no evidence of an increased risk of sICH among carefully selected patients with recent DOAC ingestion undergoing IVT compared with non-anticoagulated controls (6). Most recently, Yaghi et al. reported favorable outcomes among selected DOAC-treated patients receiving IVT, without detecting a clinically meaningful increase in hemorrhagic complications (1). Nevertheless, the observational design precludes firm conclusions regarding comparative safety or treatment effectiveness.

Recent reports from comprehensive stroke centers provide additional, although still observational evidence relevant to this evolving perspective (8). They emphasize protocol-driven patient selection, rapid assessment of anticoagulant activity, and individualized treatment decisions rather than reliance on arbitrary time-based exclusion criteria. Likewise, preliminary observational data comparing tenecteplase and alteplase in DOAC-treated patients have not identified a clear difference in hemorrhagic risk (9). These findings are hypothesis-generating, however, and are insufficient to establish the comparative safety of tenecteplase in this population.

Although these studies are observational and vulnerable to selection bias, confounding by indication, and differences in patient-selection strategies, their findings are broadly consistent across several registries and healthcare systems. Collectively, they have not demonstrated a marked increase in hemorrhagic complications that has traditionally been suspected. Nevertheless, the risk attributable to IVT cannot be determined reliably from the available evidence, and the apparently reassuring outcomes may partly reflect careful selection of patients considered at comparatively low bleeding risk. Taken together, these observations raise questions about the uniform exclusion of all patients with recent DOAC exposure from thrombolysis. They provide a rationale for evaluating more individualized approaches in which timing of the last DOAC dose, renal function, measured anticoagulant plasma levels/activity, stroke severity, and expected treatment benefit are considered comprehensively. Such an approach, however, requires prospective validation before it can broadly be incorporated into clinical practice. These Class III observational data provide preliminary reassurance regarding IVT not only after dabigatran reversal and suggest that outcomes may also be comparable in selected patients treated *without* reversal. They do not, however, establish safety or effectiveness of either strategy, and randomized evidence remains lacking. Exploring the biological basis of these observations may help determine whether the reported clinical findings are pharmacologically plausible. Mechanistic considerations cannot, however, exclude selection bias or substitute for prospective comparative evidence.

### 2.2 Biological hypotheses potentially underlying the observed clinical findings

Experimental and ex vivo studies provide several hypotheses that may be relevant to the outcomes observed in DOAC-treated patients receiving IVT, although none has been shown to account for the apparent safety of IVT in clinical AIS populations. Dabigatran directly inhibits thrombin and may influence clot architecture. Experimental and ex vivo observations suggest that dabigatran may alter fibrin-network architecture and clot susceptibility to fibrinolysis (10). These findings raise the hypothesis that thrombi formed during dabigatran treatment could be more amenable to fibrinolysis. However, this has not been directly demonstrated in thrombi retrieved from DOAC-treated patients with AIS, nor has enhanced clot lysability been shown to improve reperfusion or functional outcomes following IVT in this population.

The biological effects of factor Xa inhibitors may differ from those of dabigatran. By reducing thrombin generation, factor Xa inhibitors alter coagulation without directly inhibiting thrombin itself or platelet function. Experimental studies have also suggested that reduced thrombin generation may attenuate thrombin-mediated endothelial activation and inflammatory signaling (11, 12). Although these effects could theoretically influence neurovascular integrity, there is currently no direct clinical evidence that they reduce thrombolysis-associated intracranial hemorrhage or explain the reassuring safety signals reported in observational studies.

These mechanistic observations should therefore be regarded as hypothesis-generating rather than evidence supporting clinical safety of IVT in patients with recent DOAC exposure. They provide a biological rationale for further translational investigation but cannot resolve the effects of selection bias and confounding in existing observational cohorts or substitute for prospective clinical evidence.

### 2.3 Reversal before thrombolysis: current evidence and remaining uncertainties?

Growing evidence also prompts reconsideration of reversal strategies. For dabigatran-treated patients, idarucizumab has become widely adopted prior to IVT in many centers. Available observational evidence provides preliminary reassurance regarding this approach and indicates that it can facilitate access to IVT (5, 13, 14). However, the absence of excess hemorrhagic complications among selected dabigatran-treated patients undergoing IVT without reversal raises an important question: is reversal always necessary? Although idarucizumab has not demonstrated clinically significant prothrombotic effects, experimental data indicate that its addition to plasma samples may increase thrombin generation, raising theoretical concerns that warrant further investigation (15).

This discussion largely pertains to dabigatran because idarucizumab is the only widely available specific reversal agent in routine stroke practice. For factor Xa inhibitors, routine reversal with andexanet alfa before IVT has not been recommended by major stroke guidelines (2). Consequently, treatment decisions in selected patients generally rely on clinical assessment and, where available, measurement of residual anticoagulant activity. The availability of a reversal agent may therefore influence treatment decisions more than the underlying evidence itself. As data continue to accumulate, future practice may increasingly focus on measured anticoagulant activity rather than routine pharmacological reversal. Despite growing optimism, important challenges remain before evidence-informed IVT of patients with recent DOAC ingestion can be widely adopted. These barriers extend beyond uncertainty regarding hemorrhagic risk. They include unequal access to specialized laboratory and neurological expertise, uncertainty regarding the clinical and economic value of reversal, and substantial variation in institutional workflows. Table 1 summarizes these challenges and identifies potential strategies for more consistent and evidence-informed implementation.

**Table 1:** Current barriers and future opportunities for evidence-informed intravenous thrombolysis in patients with recent direct oral anticoagulant exposure.

Challenge	Clinical implications	Potential solutions
Limited availability of DOAC-specific assays	Delays treatment or prevents informed decision-making	Wider implementation of rapid anti-Xa and dabigatran-specific testing
Variable laboratory turnaround times	Reduced eligibility within narrow therapeutic windows	Point-of-care or expedited emergency laboratory pathways
Uncertainty regarding safe anticoagulant thresholds	Inconsistent treatment decisions across centers	International consensus and prospective validation studies
Heterogeneity of existing observational studies	Limited generalizability	Large multicenter registries and standardized reporting
Persistent guideline conservatism	Underutilization of reperfusion therapies	Guideline updates incorporating contemporary evidence
Differences between comprehensive and community stroke centers	Unequal access to specialist assessment, drug-specific testing, and advanced decision-making pathways	Simplified regional protocols, expedited consultation pathways, and telemedicine support
Uncertain role of reversal agents	Potential overtreatment, workflow delays, additional costs, and exposure to agent-specific risks without established incremental benefit	Comparative-effectiveness studies of reversal-based, assay-guided, and non-reversal strategies, including cost-effectiveness analyses

### 2.4 Future directions: from observational evidence to individualized reperfusion

Despite increasingly reassuring clinical evidence, several uncertainties must be addressed before IVT in patients with recent DOAC exposure can be more broadly incorporated into clinical practice and guideline recommendations.

#### 2.4.1 Prospective evidence and standardized data collection

Current evidence is derived almost exclusively from observational studies and registries (1, 5–9, 13, 14). Although their findings are broadly consistent, residual confounding, selection bias, and confounding by indication remain important limitations. Patients selected for IVT are often those perceived to have a lower hemorrhagic risk, restricting the generalizability of reported outcomes. Randomized trials would provide the most reliable evidence, and the results of investigations such as DO-IT and PASSION are therefore important (16, 17). Nevertheless, recruitment may prove challenging if clinicians become increasingly reluctant to withhold potentially beneficial treatment from carefully selected patients. Prospective multicenter registries, standardized definitions and reporting, and appropriately applied causal-inference methods will consequently remain valuable complements to randomized evidence.

#### 2.4.2 Anticoagulant activity-guided selection

Reliance on the interval since the last DOAC dose provides only an indirect estimate of anticoagulant activity and does not account for variability in absorption, renal clearance, age, comorbidities, treatment adherence, or concomitant medications. Future studies should therefore determine whether drug-specific measurements can more reliably identify patients with minimal or absent anticoagulant activity.

Calibrated anti-Xa assays provide the most specific routinely available approach for factor Xa inhibitors, whereas diluted thrombin time and ecarin-based assays can assess dabigatran activity. Their clinical utility is currently limited by restricted availability, variable calibration, uncertain treatment thresholds, and turnaround times that may exceed the narrow therapeutic window for IVT. Rapid emergency laboratory pathways and point-of-care platforms could facilitate treatment decisions, but their analytical performance, clinically relevant thresholds, and effects on treatment times and outcomes require prospective validation. Until such evidence is available, laboratory results should be interpreted alongside the timing of last dose, renal function, stroke characteristics, imaging findings, and anticipated benefit of treatment.

Implementation may be especially difficult in community hospitals without drug-specific assays or on-site vascular neurology expertise. Simplified regional protocols and telemedicine consultation could support standardized assessment and reduce disparities between community and comprehensive stroke centers. However, telemedicine cannot compensate for unavailable or delayed laboratory testing, and its effectiveness in guiding IVT decisions in this setting has not been prospectively established.

#### 2.4.3 Defining the role of reversal agents

The appropriate role of reversal before IVT also remains uncertain. Observational data provide preliminary reassurance regarding idarucizumab followed by IVT in dabigatran-treated patients (7, 13, 14). However, reports of comparable outcomes among selected patients treated without reversal raise the possibility that idarucizumab may not be necessary when residual dabigatran activity is minimal or absent (6, 7). These non-randomized comparisons cannot establish the relative safety of either strategy.

Routine reversal may also expose patients to an additional intervention when clinically relevant anticoagulant activity is no longer present, potentially increasing costs and workflow complexity without established incremental benefit. Future studies should therefore compare reversal-based, assay-guided, and carefully selected non-reversal strategies, including their effects on hemorrhagic and thrombotic complications, treatment delays, functional outcomes, and cost-effectiveness.

For factor Xa inhibitors, routine reversal before IVT has not been endorsed by major stroke guidelines (2). Progress in this area is more likely to depend on reliable measurement of residual anticoagulant activity than on routine reversal. The principal research priority is therefore to establish whether validated drug-specific thresholds can identify patients in whom IVT may be considered with an acceptable benefit–risk balance.

#### 2.4.4 Toward individualized reperfusion decisions

Recent DOAC exposure is only one component of a complex treatment decision that also includes age, stroke severity, infarct size, renal function, blood pressure, baseline functional status, and imaging characteristics. Future prediction models may integrate these variables with drug-specific anticoagulant activity to estimate the expected benefit and hemorrhagic risk of IVT for individual patients.

Automated imaging analysis and artificial intelligence-assisted decision support may eventually facilitate this approach, while imaging markers of blood–brain barrier disruption or microvascular injury could provide additional risk information. These strategies remain investigational, however, and require external validation, assessment of clinical utility, and evaluation across different healthcare settings. The immediate priority is therefore not to replace existing eligibility criteria with unvalidated prediction tools, but to generate prospective evidence necessary for transparent and reproducible individualized decision-making.

## 3 Discussion

Available observational evidence suggests that recent DOAC exposure alone may be an imprecise surrogate for sICH risk. Across several registries and multicenter cohorts, selected DOAC-treated patients receiving IVT have not shown a clear excess of sICH, and some studies have reported associations with more favorable functional outcomes (1, 5–9, 13, 14). These findings are clinically important but should not be interpreted as establishment of IVT safety or effectiveness in all patients with recent DOAC intake. Selection bias, residual confounding, and confounding by indication remain plausible explanations for at least part of the apparently reassuring results.

Accordingly, available evidence does not justify indiscriminate treatment or uniform deviation from current guideline recommendations. It does, however, provide a rationale for reconsidering whether fixed time-based exclusion criteria adequately reflect heterogeneity of residual anticoagulant activity and individual treatment benefit. The relevant clinical question is therefore not simply whether recent DOAC exposure is present, but whether clinically meaningful anticoagulant activity is likely to persist and whether the anticipated benefit of IVT outweighs the estimated bleeding risk.

Prospective studies, including investigations such as DO-IT and others (16), remain essential. Priorities include validation of drug-specific anticoagulant thresholds, comparison of reversal-based and non-reversal strategies, development of rapid laboratory pathways, and standardized reporting across healthcare systems. Until stronger evidence is available, decisions should remain cautious and individualized, incorporating the timing of the last dose, renal function, drug-specific testing when available, clinical and imaging characteristics, and current guideline recommendations. This approach acknowledges the limitations of the existing evidence while permitting continued evaluation of whether selected patients with recent DOAC exposure may reasonably be considered for IVT.

## Data Availability

The original contributions presented in the study are included in the article/supplementary material, further inquiries can be directed to the corresponding author.
